# Electrical Resistance Behavior of Printed Ag Nanowire Electrode Lines Under Transverse Tensile Strain

**DOI:** 10.3390/nano16201333

**Published:** 2026-10-21

**Authors:** Yoon Jae Moon, Jiyeong Kong, Sukkee Um, Kyung-Tae Kang

**Affiliations:** 1 Autonomous Manufacturing & Process R&D Department, Korea Institute of Industrial Technology, 143 Hanggaul-ro, Sangnok-gu, Ansan-si 15588, Republic of Korea; myj3235@kitech.re.kr (Y.J.M.);; 2 School of Mechanical Engineering, Hanyang University, 222 Wangsimni-ro, Seongdong-gu, Seoul 04763, Republic of Korea

**Keywords:** silver nanowires, printed stretchable electrodes, transverse tensile strain, electrical resistance, conductive network damage

## Abstract

Printed stretchable interconnects may experience tensile deformation perpendicular to the direction of current flow, but their electrical response under transverse loading remains insufficiently understood. In this study, dispenser-printed silver nanowire (Ag NW) line electrodes on polydimethylsiloxane were subjected to transverse tensile strains of 10–40% for 10 stretching–release cycles. Ten specimens were tested at each strain level. Resistance was measured in situ, and deformation-induced changes in the Ag NW network were examined using optical microscopy and field-emission scanning electron microscopy (FESEM). To distinguish the effects of macroscopic deformation from changes in network connectivity, the measured resistance was compared with geometric predictions based on Poisson-induced shortening of the printed line. At 10% strain, the stretched-state resistance closely followed the geometric prediction and remained nearly unchanged during cycling. At 20% strain, the resistance increased progressively with repeated cycling and local cracks were observed, whereas the first-cycle deviation from the geometric reference remained small relative to the specimen-to-specimen variation. At 30% and 40% strain, the measured resistance clearly exceeded the geometric reference in both the stretched and released states. After 10 cycles at 40% strain, the corresponding relative resistances reached 1.369 and 1.736, respectively. FESEM observations showed that conductive nanowire bridges remained across locally cracked regions at 20% strain, whereas crack penetration into the line interior and local pathway loss were observed at 30% strain. These results indicate an intermediate response at 20% strain and a clear transition to a network-damage-dominated response at 30% strain.

## 1. Introduction

Stretchable electronics have attracted considerable interest for applications such as deformable displays [1,2], wearable devices [3], smart textiles [4], and flexible photovoltaic systems [5]. Reliable operation of these devices requires conductive electrodes that maintain continuous electrical pathways under repeated and multiaxial deformation. Carbon-based nanomaterials, particularly carbon nanotubes (CNTs) and graphene, have been widely investigated for stretchable electrodes and piezoresistive sensors. CNT networks can accommodate large deformation through changes in nanotube contacts, bundle separation, and network rearrangement [6,7], while graphene-based networks exhibit strain-dependent resistance through structural deformation and changes in percolation pathways [8,9]. However, the electrical responses of these carbon-based networks are strongly influenced by contact resistance and the disruption or rearrangement of conductive pathways under strain. Ag nanowire (Ag NW) networks offer high electrical conductivity, network-level mechanical compliance, and compatibility with low-temperature solution processing, making them particularly suitable for printed stretchable electrodes [10,11]. Nevertheless, repeated deformation can cause nanowire sliding, junction separation, and crack formation, resulting in irreversible changes in the conductive network and electrical resistance.

Printed stretchable interconnects rarely experience purely longitudinal deformation during manufacturing or service. Transverse strain can arise when line electrodes are attached to curved surfaces, integrated into multilayer devices, or placed on skin and soft structures undergoing multiaxial deformation [1,2,3,4,5,12,13,14,15,16]. Transverse deformation may also occur during manufacturing processes such as roll-to-roll transport and lamination. This deformation mode is mechanically and electrically distinct from longitudinal stretching [17,18,19,20]. When a line is stretched along the current-flow direction, the increased line length and progressive disruption of nanowire junctions generally increase resistance [21,22,23,24,25,26,27]. Under transverse tensile strain, however, Poisson contraction of the substrate shortens the line along the current-flow direction and tends to decrease resistance, whereas nanowire separation, crack formation, and junction loss tend to increase it [19,22,28,29]. These opposing contributions can produce little apparent change in the measured resistance even while irreversible network damage accumulates. Transverse stretching is therefore important for reliability assessment because an apparently stable resistance in the stretched state does not necessarily indicate that the electrode network remains intact.

Previous studies have addressed related directional effects, although not the same printed-line configuration examined here. Biaxially stretchable Ag NW conductors retained conductivity by using buckled or wavy networks, but their emphasis was the attainable multiaxial stretchability of continuous transparent films rather than resistance evolution along a narrow printed line [18]. Network-level microscopy and electrical measurements subsequently showed that anisotropy strongly changes the response measured parallel and perpendicular to the loading direction and identified tensile fracture, compressive kinking, and constrained bending as orientation-dependent nanowire deformation modes [19]. Printed Ag NW electrodes have also exhibited strain-induced rotation and alignment, demonstrating that wire mobility and substrate adhesion can change the resistance in the released state [20]. Directly printed Ag NW strain sensors have primarily been evaluated using resistance changes produced by strain along the sensing direction [12]. More recent aligned-network studies likewise confirm that electrical resistance and strain sensitivity depend on the angle between preferred nanowire orientation, loading direction, and current flow [17,30]. These studies establish that transverse and orientation effects exist; however, they do not isolate the competing resistance-lowering effect of Poisson-induced line shortening from the resistance-raising effect of junction and crack damage in a printed line. Recent advances have improved Ag NW electrode performance through ultralong nanowires, junction welding, printable ink formulations, semi-embedded architectures, deep-printed interconnects, and shear-aligned composites [31,32,33,34,35,36,37]. These studies focus mainly on network fabrication and structural design rather than the state-resolved electrical response of a narrow printed line under transverse loading.

Accordingly, this study investigates the electrical response and damage evolution of dispenser-printed Ag NW lines on PDMS under transverse tensile strain. Whereas our previous study [20] examined nanowire alignment, substrate adhesion, and released-state resistance under strain applied parallel to the printed line, the present work applies strain perpendicular to the line and measures resistance separately in the stretched and released states. By comparing these measurements with a Poisson-based geometric reference and morphological observations, we distinguish reversible line shortening from irreversible crack- and junction-related changes in the conductive network.

## 2. Materials and Methods

### 2.1. Materials and Fabrication of Ag NW Electrode Lines

The Ag NW ink (DSNWI-20, DS Hi-metal, Ulsan, Republic of Korea) consisted of 0.5 wt% silver nanowires with an average diameter of 25 nm and a length of 25 μm, dispersed in deionized water. The ink was printed onto a 250-μm-thick PDMS film (PS10-250, AMED, Gunpo, Republic of Korea) using a drop-on-demand dispenser (MDV3200A, Vermes, Holzkirchen, Germany) operated by a piezoelectric actuator and equipped with a 70-μm nozzle. Before printing, the PDMS film was treated with UV/O_3_ for 25 min to produce a hydrophilic surface. The printed diameter of a single droplet was approximately 580 μm, and the center-to-center distance between droplets was 300 μm. The printed Ag NW electrode lines were 40 mm × 680 μm (length × width). After printing, the lines were dried under ambient conditions for 10 min and then sintered at 100 °C for 30 min. Liquid metal (EGaIn, Merck, Darmstadt, Germany) and copper wires were used as electrical contacts for real-time measurements during stretching and were encapsulated with PDMS for mechanical fixation. To quantify resistance variation originating from the contacts, copper-wire/EGaIn assemblies were tested without a Ag NW line. Three independent control measurements were conducted at each strain level, with 10 stretching–release cycles per measurement. The maximum absolute resistance change was determined at each strain. To express its possible contribution on the same R/R_0_ scale as the Ag NW measurements, this change was divided by 110 Ω, the lower bound of the initial Ag NW resistance range of 110–140 Ω. This conservative calculation gave shaded-envelope half-widths of ±0.048%, ±0.536%, ±0.529%, and ±2.673% at 10%, 20%, 30%, and 40% strain, respectively. These values represent the maximum observed control-system variation and are not statistical confidence intervals.

### 2.2. Electromechanical Testing and Morphological Characterization

**Figure 1:**
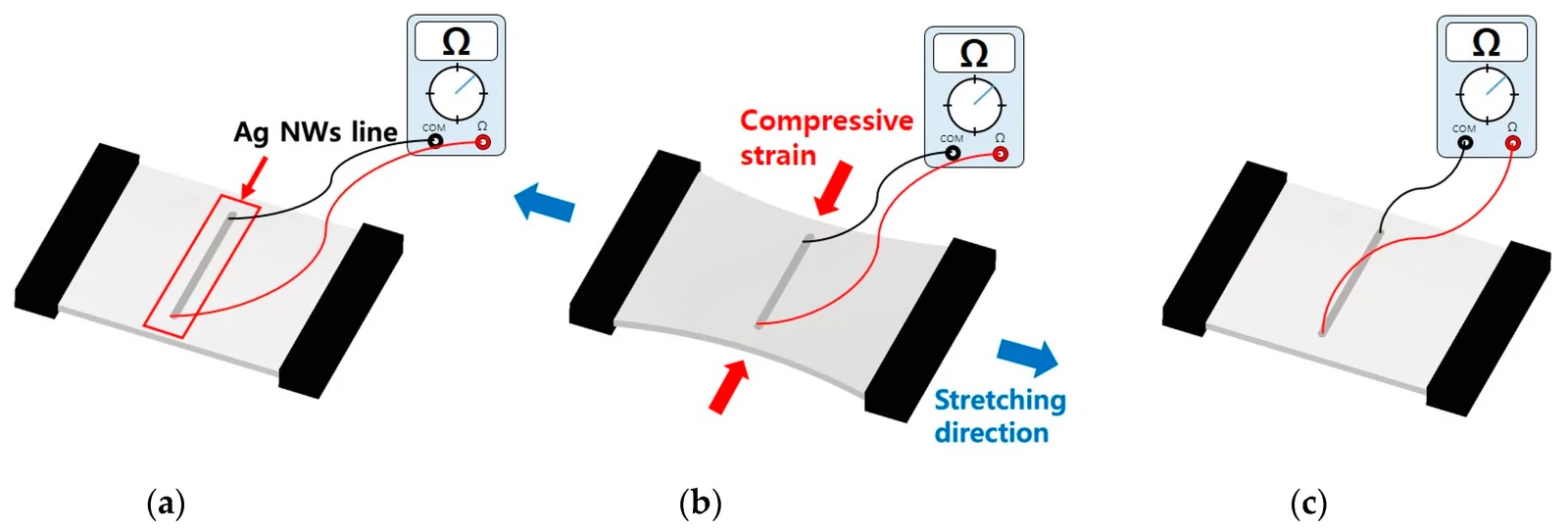
Schematic illustration of the transverse stretching test of a printed Ag NW electrode line: (**a**) initial state, (**b**) stretched state, and (**c**) released state.

**Figure 2:**
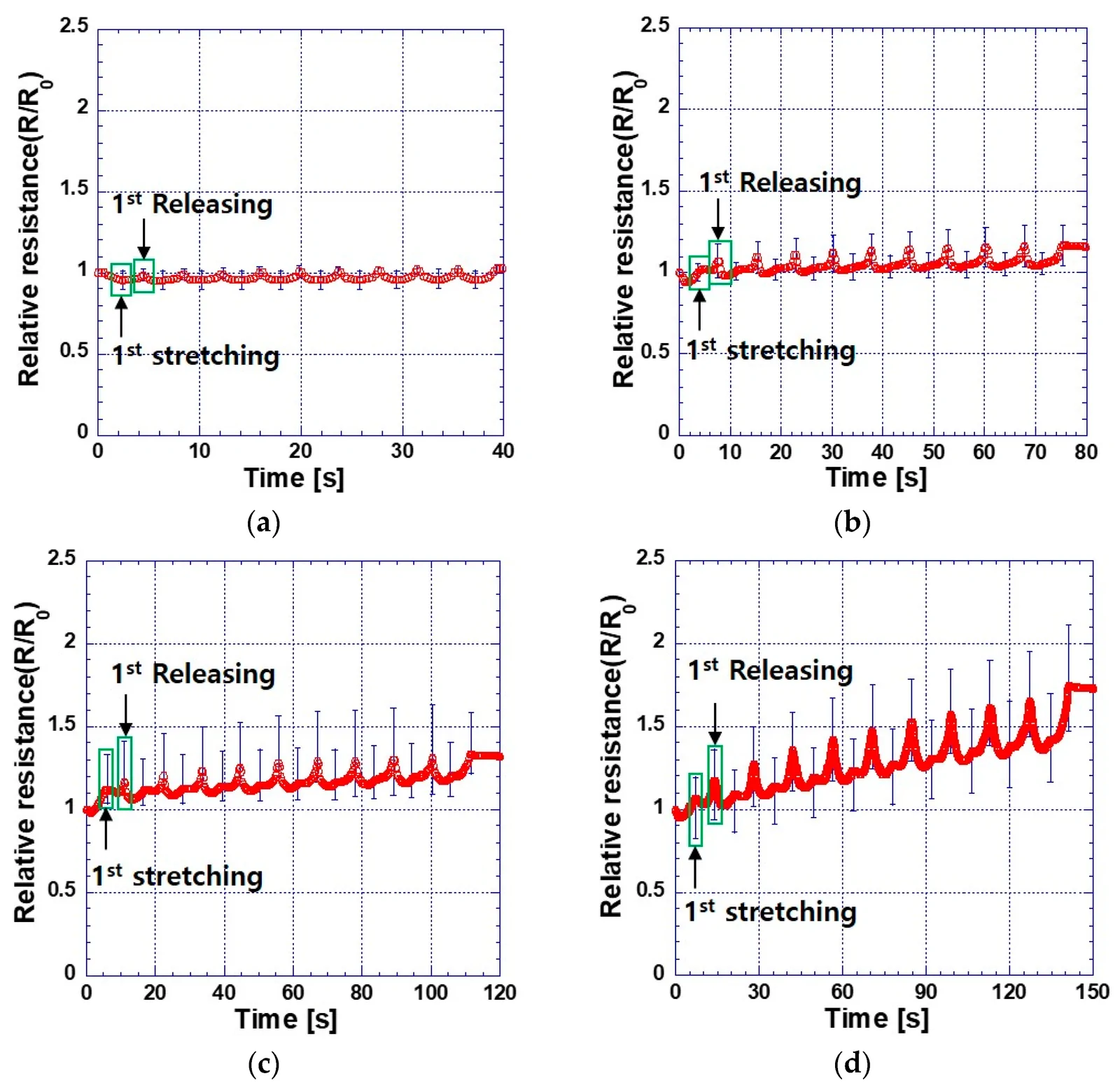
Mean relative resistance of printed Ag NW line electrodes during 10 transverse stretching–release cycles at (**a**) 10%, (**b**) 20%, (**c**) 30%, and (**d**) 40% strain (10 specimens per strain level). Red circles represent the mean relative resistance of 10 specimens, and blue error bars indicate ±1 standard deviation.

The Ag NW electrode line printed on the PDMS film was stretched transverse to its longitudinal axis. As illustrated in Figure 1, transverse tensile strain induces longitudinal compressive strain through the Poisson effect, thereby reducing the electrode length. Tests were performed at transverse strain levels of 10%, 20%, 30%, and 40%. Ten independently prepared specimens were tested at each strain level, and each specimen underwent 10 stretching–release cycles. During the cyclic tests, the longitudinal electrical resistance was measured in situ using a digital multimeter (34461A, Keysight, Santa Rosa, CA, USA) at 0.3-s intervals. Relative resistance was expressed as R/R_0_, where R is the resistance in the stretched or released state. For each specimen, R_0_ was measured before the first application of strain and used as the common normalization reference for all cycles. Figure 2 presents the pointwise ensemble-averaged resistance histories. The strain-dependent Poisson’s ratio of the PDMS film was determined from the ratio of transverse contraction to longitudinal extension using 50 mm × 82 mm specimens. The Poisson’s ratio was calculated using Equation (1):

(1) $$\nu =-\frac{\varepsilon_{\mathrm{long}}}{\varepsilon_{\mathrm{trans}}}$$

where ν is Poisson’s ratio and *ε_long_* and *ε_trans_* are the longitudinal and transverse strains, respectively. The Poisson’s ratio of the PDMS film at the applied strain levels is summarized in Table 1.

The morphological evolution of the Ag NW electrode in the stretched and released states was observed using an optical microscope (DZ2, Union, Japan). Microstructural changes in the Ag NW network were examined using FESEM (SU8010, Hitachi, Japan). A custom-designed stretching jig that enabled observation in the stretched state was used for FESEM analysis.

ChatGPT (OpenAI; image generation feature; version not recorded) was used solely to prepare the graphical abstract. The generated image was reviewed and edited by the authors.

## 3. Results and Discussion

### 3.1. Electrical Response Under Transverse Tensile Strain

Figure 2 shows the mean relative resistance of the printed Ag NW line electrodes during 10 transverse stretching–release cycles.

Ten specimens were tested at each strain level, and the data in Figure 2 represent their pointwise mean relative resistance; the numerical values below are reported as mean ± standard deviation. At all strain levels, the resistance measured in the stretched state was lower than that measured after release. At 10% strain (Figure 2a), the stretched-state resistance remained nearly constant, changing from 0.957 ± 0.029 in the first cycle to 0.962 ± 0.030 in the tenth cycle. The released-state resistance increased slightly from 0.975 ± 0.027 to 1.027 ± 0.013 over the same cycles. Thus, both the stretched- and released-state resistances remained close to their initial values during the 10 cycles. At 20% strain (Figure 2b), the stretched-state resistance increased from 0.944 ± 0.038 in the first cycle to 1.042 ± 0.043 in the tenth cycle. The released-state resistance increased from 1.050 ± 0.037 to 1.170 ± 0.071. The resistance therefore increased progressively with repeated cycling, and the increase was more pronounced after release. At 30% strain (Figure 2c), the stretched-state resistance increased from 0.984 ± 0.021 to 1.172 ± 0.093, while the released-state resistance increased from 1.143 ± 0.079 to 1.334 ± 0.110. At 40% strain (Figure 2d), the stretched- and released-state resistances reached 1.369 ± 0.171 and 1.736 ± 0.217, respectively, by the tenth cycle. These results show that the resistance increase became larger with increasing strain and repeated cycling. The released-state resistance also remained higher than the stretched-state resistance, with the difference between the two states becoming more pronounced at higher strain levels. Optical images from the first stretched and released states are provided in the Appendix A.

### 3.2. Morphological Evolution and Network Damage

**Figure 3:**
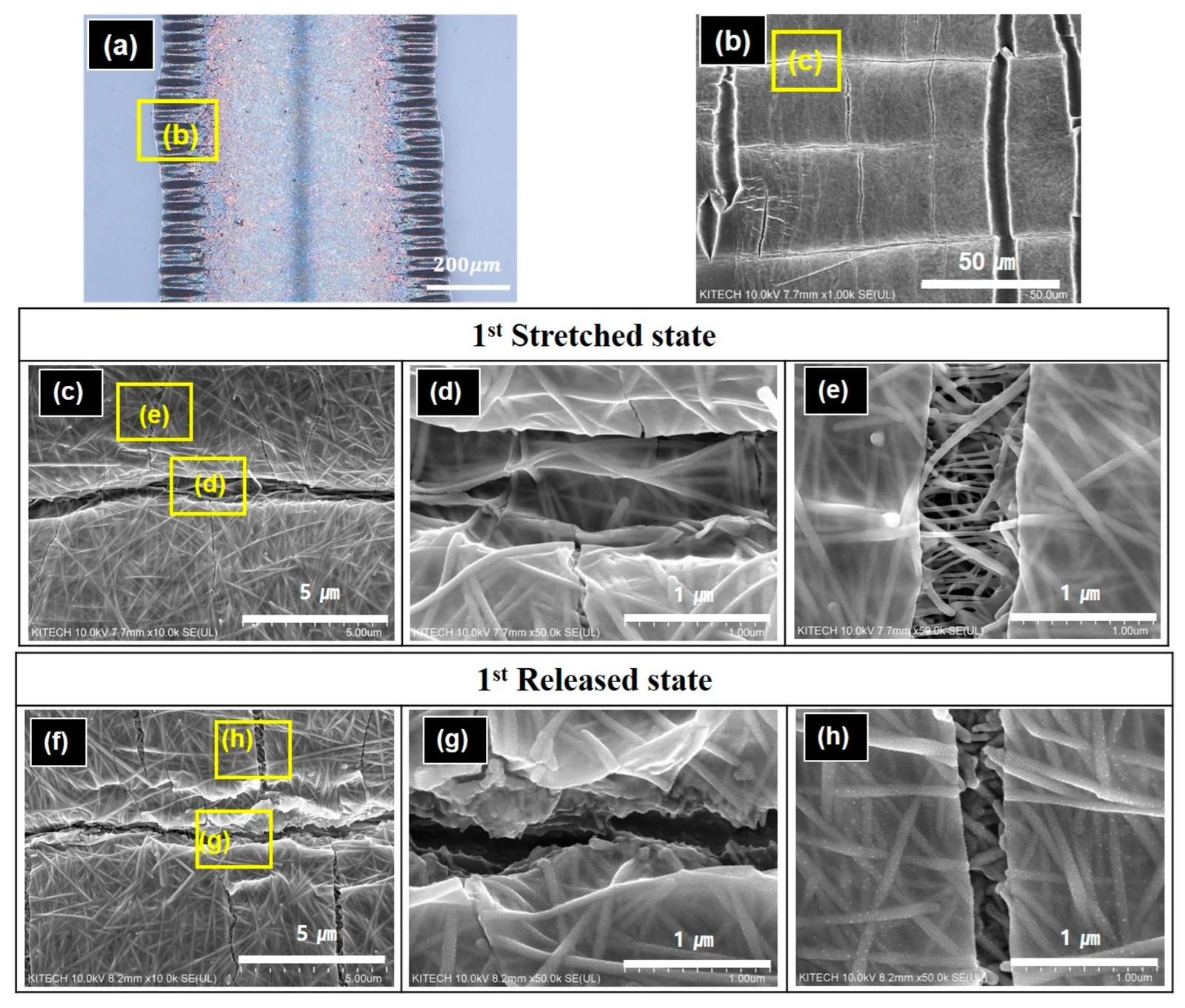
Optical and FESEM images of a Ag NW line at 20% transverse strain: (**a**) optical overview of the stretched line; (**b**) low-magnification FESEM image of the selected region; (**c**–**e**) successively enlarged images in the first stretched state; and (**f**–**h**) corresponding images after the first release. Panels (**d**,**g**) show a region with nanowire separation, whereas panels (**e**,**h**) show a region in which nanowire bridges remain across the crack. Yellow boxes indicate the regions enlarged in the subsequent panels.

Figure 3 presents optical and FESEM observations of the Ag NW line in the stretched and released states at 20% transverse strain.

**Figure 4:**
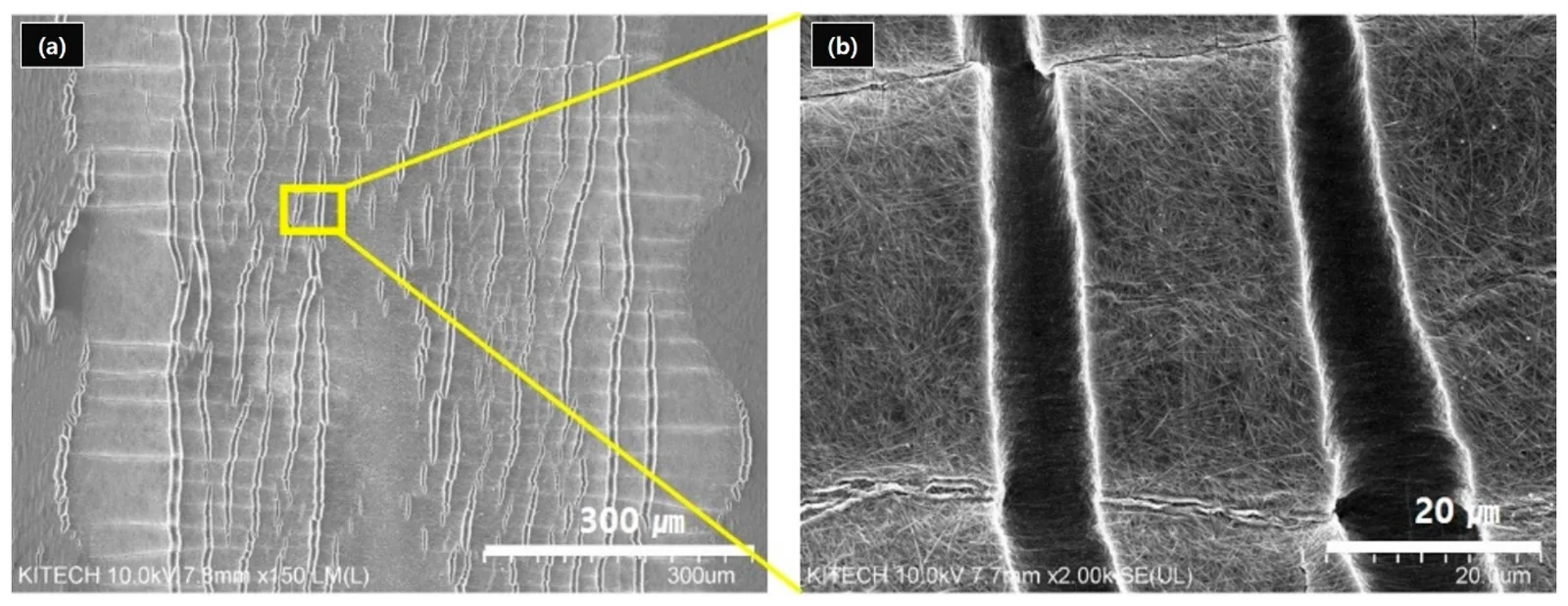
FESEM images of a Ag NW line in the stretched state at 30% transverse strain: (**a**) low-magnification overview showing cracks extending from the line edge toward the interior and (**b**) enlarged view of the boxed region showing crack penetration and local loss of network continuity. The yellow box indicates the enlarged region.

At 20% transverse strain, local crack formation was accompanied by changes in the conductive pathways of the Ag NW network. In Figure 3d, several Ag NWs above a crack are separated in the stretched state, whereas nanowires beneath the crack continue to bridge its two sides. These residual connections may help limit the resistance increase during stretching. After release, additional separation of the same bridging nanowires is visible (Figure 3g), consistent with the higher released-state resistance. Other locally cracked regions retain visible connections in both the stretched and released states (Figure 3e,h), suggesting that the electrical response depends more strongly on the retention of conductive bridges than on the apparent crack orientation alone. At 30% strain (Figure 4), the cracks extend from the line edge toward the interior and are accompanied by more extensive junction loss. Such crack penetration can reduce the effective conductive width and eliminate parallel current paths, consistent with the larger resistance increase observed at this strain. Overall, the microstructural observations support a qualitative relationship between resistance growth, crack penetration, and the loss of nanowire bridges. A quantitative correlation between individual crack characteristics and resistance, however, cannot be established from the selected local FESEM observations.

### 3.3. Geometric and Network Contributions to the Electrical Resistance

When tensile strain is applied perpendicular to a printed electrode line, Poisson contraction shortens the current path and tends to decrease resistance. In a Ag NW electrode, however, conduction depends on percolating pathways through nanowires and nanowire–nanowire junctions [21,23,24,25,26]. Transverse deformation can increase local inter-nanowire separation, raise junction resistance, and disrupt continuous pathways. Electron tunneling may contribute across small gaps before complete disconnection because tunneling resistance increases strongly with separation distance [38,39,40]. Since nanoscale gaps were not measured, tunneling is treated only as a plausible contribution rather than a quantitatively verified mechanism. The resistance of a continuous electrode line can be expressed as

(2) $$R=\rho \frac{L}{W\cdot t}$$

where *ρ*, *L*, *W*, and *t* denote the resistivity, length, width, and thickness of the electrode, respectively. For the initial and deformed states, the relative resistance is therefore written as

(3) $$R_{\mathrm{eff}}\propto \rho \frac{L_{\mathrm{eff}}}{W_{\mathrm{eff}}}$$

where *ρ_eff_* and *W_eff_* are effective quantities representing the electrical response of the deformed nanowire network. In particular, *W_eff_* denotes the portion of the printed line that maintains continuous conductive pathways. It may decrease when cracks penetrate the line or nanowire junctions become disconnected, even if the macroscopic line width does not decrease. Equation (3) identifies the factors that can influence the measured resistance, but it is not intended as a junction-resolved percolation model because *ρ_eff_* and *W_eff_* were not measured independently.

The applied transverse strain is defined as

(4) $$\varepsilon_{\mathrm{trans}}=\frac{∆W}{W_{0}}$$

and the strain induced along the printed line is

(5) $$\varepsilon_{\mathrm{long}}=\frac{∆L}{L_{0}}=-\nu \varepsilon_{\mathrm{trans}}$$

where ν is the strain-dependent Poisson’s ratio of the PDMS substrate. The length of the stretched electrode is consequently given by

L = *L*_0_(1 + *ε_long_*) = *L*_0_(1 − *νε_trans_*) (6)

To establish a geometric reference, changes in intrinsic resistivity, effective conductive width, and thickness are initially neglected. Under the restricted assumptions that *ρ_eff_* ≈ *ρ*_0_, *W_eff_* ≈ *W*_0_, and *t* ≈ *t*_0_, Equation (3) becomes

(7) $$\frac{R}{{\;R}_{0}}\approx \frac{L}{L_{0}}=1-\nu \varepsilon_{\mathrm{trans}}$$

Equation (7) represents the relative resistance expected from Poisson-induced shortening of the current path when the conductive network remains intact. At each strain level, Equation (7) was evaluated using the corresponding strain-dependent Poisson’s ratio listed in Table 1 (ν = 0.37, 0.37, 0.32, and 0.30 at 10%, 20%, 30%, and 40% strain, respectively), rather than a single fixed value. It therefore serves as a geometric reference and does not account for resistance increases caused by crack formation or junction disconnection. For comparison, the resistance change predicted using a conventional bulk-geometric model is expressed as

(8) $$\frac{R}{R_{0}}=\frac{1}{1+\varepsilon_{\mathrm{trans}}}$$

**Figure 5:**
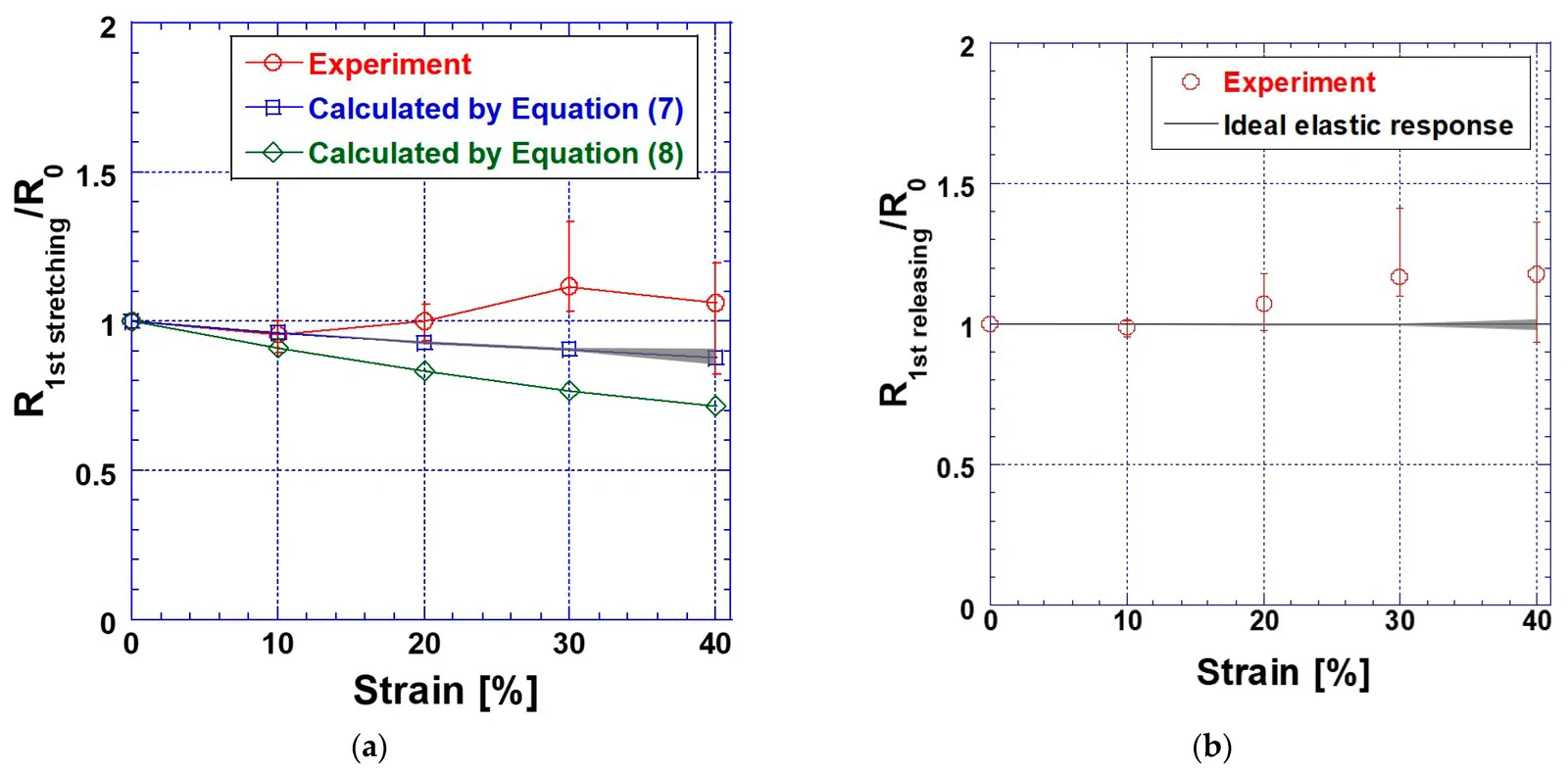
Comparison between the experimentally measured and analytically calculated relative resistance of dispenser-printed Ag NW lines under transverse tensile strain: (**a**) first-cycle relative resistance in the stretched state compared with the geometric reference calculated from Equation (7), and (**b**) first-cycle relative resistance in the released state compared with the ideal recovery reference, *R*/*R*_0_ = 1. The data represent the mean of 10 independently prepared specimens at each strain level, and the error bars show the observed minimum-to-maximum range among the specimens. The gray shaded envelopes show the strain-specific empirical bounds of the copper-wire/EGaIn measurement system, defined from the maximum absolute resistance change after stabilization across three independent control measurements and expressed on the Ag NW *R*/*R*_0_ scale using a conservative 110 Ω reference.

In the released state, recovery of the macroscopic line length gives a geometric reference of *R*/*R*_0_ = 1. A released-state resistance greater than unity therefore indicates that the initial electrical condition was not fully recovered. Figure 5 compares the first-cycle mean relative resistance in the stretched and released states with the corresponding geometric references. The same specimen-specific R_0_ values defined in Section 2.2 were used for normalization. The first stretched- and released-state values were extracted separately for each specimen, and the error bars show the observed minimum-to-maximum range among the 10 specimens. The strain-specific contact-system variations determined using the control assemblies described in Section 2.1 were used to define the shaded envelopes in Figure 5.

Figure 5a compares the first-cycle stretched-state resistance with the geometric reference calculated using Equation (7). At 10% strain, the measured resistance remains close to the geometric reference, indicating that Poisson-induced shortening adequately describes the response within the experimental variation. At 20% strain, the mean resistance is higher than the geometric reference and lies outside the control-system envelope. However, the difference remains small relative to the specimen-to-specimen range; therefore, no statistically resolved transition is claimed at this strain. The cycle-dependent resistance increase and local cracks observed at 20% strain are consistent with limited irreversible network changes, while the nanowire bridges shown in Figure 3 continue to maintain conductive pathways across locally cracked regions. At 30% and 40% strain, the deviation from the geometric reference becomes pronounced, and the morphological observations are qualitatively consistent with crack penetration into the line interior and conductive-pathway loss.

Figure 5b compares the first-cycle released-state resistance with the ideal recovery reference of *R*/*R*_0_ = 1. At 10% strain, the resistance returns close to *R*_0_, indicating substantial recovery of the conductive network. At 20% strain, the mean resistance is higher than *R*_0_ and lies outside the shaded control-system envelope. Together with the cycle-dependent increase and local cracks, this response suggests that limited irreversible network changes may already occur at 20% strain, although network damage does not yet dominate the overall electrical response. Larger deviations are observed at 30% and 40% strain and are qualitatively consistent with crack penetration and conductive-pathway loss. Thus, comparison with the geometric references distinguishes resistance changes associated with reversible line deformation from those accompanied by irreversible changes in the conductive network, with the first clearly damage-dominated response observed at 30% strain.

The balance between geometric deformation and conductive-network damage may vary with the substrate’s Poisson’s ratio, stiffness, viscoelasticity, and interfacial adhesion. Elastic mismatch between stiff Ag NWs and a compliant substrate can produce nonuniform strain transfer and promote junction separation, sliding, or local delamination. Our previous adhesion results support substrate-dependent network rearrangement [20]; therefore, the quantitative transition identified here is specific to the Ag NW–PDMS system. Temperature and humidity can also affect the resistance and long-term stability of Ag NW networks [41,42,43], so the present conclusions are limited to the tested laboratory conditions.

## 4. Conclusions

This study investigated the electrical response of dispenser-printed Ag NW line electrodes subjected to transverse tensile strain. The resistance measured in the stretched state was consistently lower than that measured after release, and the difference between the two states increased with strain and repeated cycling. At 10% strain, the measured response remained close to the geometric reference based on Poisson-induced shortening of the printed line. At 20% strain, resistance increased progressively with cycling and local cracks were observed, whereas the first-cycle deviation remained small relative to the specimen-to-specimen variation and was not treated as a statistically resolved transition. This condition was therefore identified as an intermediate response. At 30% and 40% strain, the deviation from the geometric reference became pronounced, and the morphological observations were qualitatively consistent with crack penetration into the line interior and conductive-pathway loss. Comparison of stretched- and released-state resistance with the corresponding geometric references demonstrates that measurements in both states are necessary to distinguish reversible line deformation from irreversible conductive-network changes. Thus, among the strain levels examined, the first clearly damage-dominated response was observed at 30% strain.

The present results are limited to the specific Ag NW–PDMS configuration and test conditions used in this study, which employed a single electrode geometry, Ag NW concentration, and strain rate under laboratory conditions. It therefore remains to be determined whether the observed electrical response and damage transition apply to other material configurations and test conditions. In addition, the FESEM observations provide qualitative evidence of network damage but do not quantify crack density, crack length, or the fraction of surviving nanowire bridges. Future studies should systematically examine the effects of substrate mechanical and interfacial properties, electrode dimensions, Ag NW concentration, strain rate, and environmental exposure, together with quantitative characterization of deformation-induced cracks. High-resolution three-dimensional techniques such as FIB-SEM could further enable quantitative analysis of the deformed conductive network [44].

## Figures and Tables

**Table 1:** Strain-dependent Poisson’s ratios of the PDMS film.

	Strain 10%	Strain 20%	Strain 30%	Strain 40%
Poisson’s ratio	0.37	0.37	0.32	0.3

**Figure A1:**
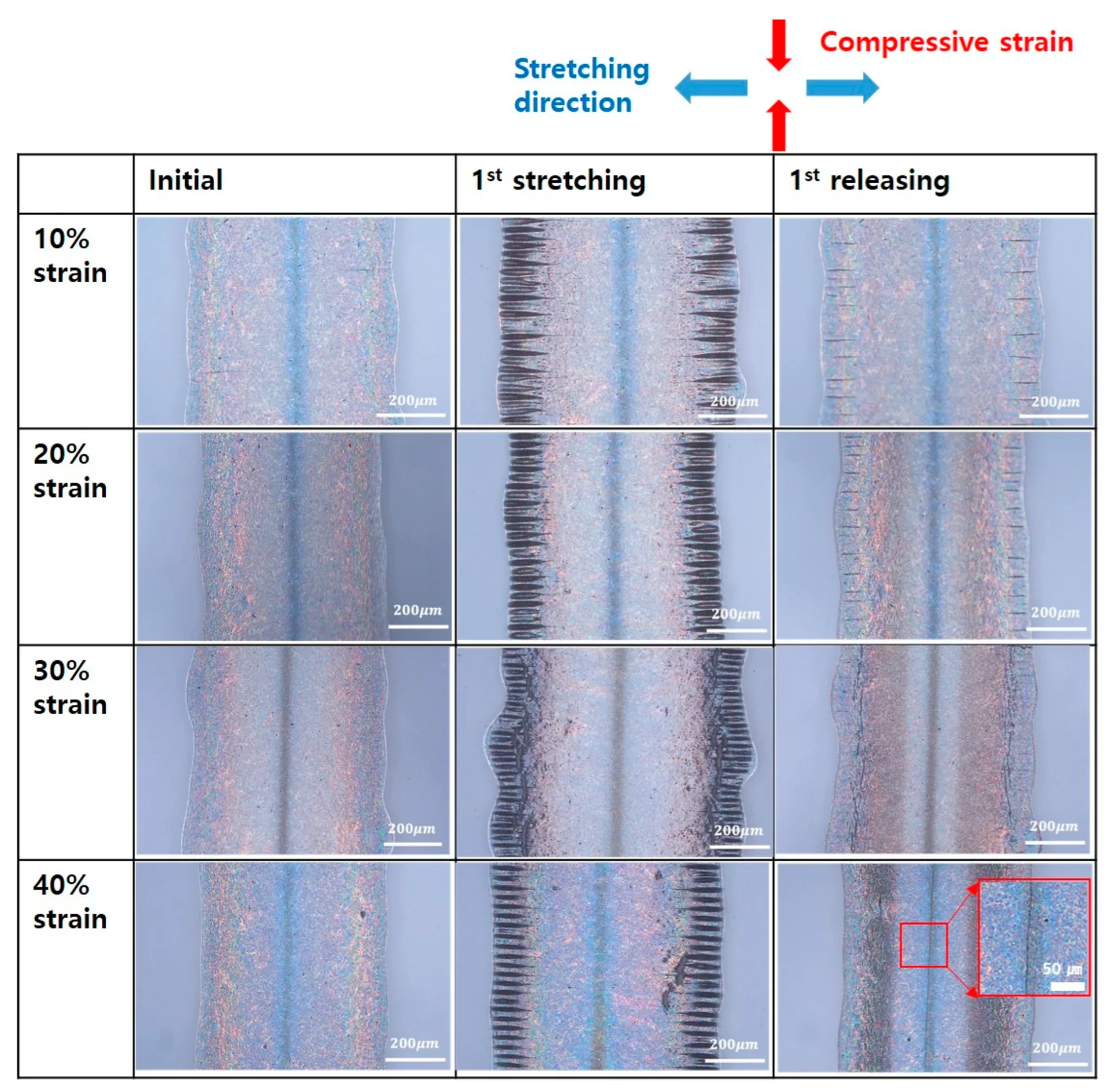
Optical micrographs of Ag NW lines in the first stretched and released states at each transverse strain. The red box marks the persistent central crack observed after unloading at 40% strain.

## Data Availability

The raw data supporting the conclusions of this article will be made available by the authors upon request.

## Appendix A

Figure A1 shows the first stretched and released states. Interior cracks at 30–40% strain and a persistent central crack at 40% strain (red box) are qualitatively consistent with the resistance changes in Figure 2; the images were not statistically sampled.
